# Experiences of restrictive practices in child and adolescent mental health inpatient care: an integrative review

**DOI:** 10.1186/s13034-026-01119-z

**Published:** 2026-07-01

**Authors:** S. U. Larsen, M. L. Pedersen, J. Baker, J. P. Hansen, S. Lemcke, F. A. Gildberg

**Affiliations:** 1 Forensic Mental Health Research Unit, Department of Regional Health Research, University of Southern Denmark, Campusvej 55, 5230 Odense M, Denmark; 2 Psychiatric Department Middelfart, Lillebaelt Hospital, Region of Southern Denmark, Østre Hougvej 70, 5500 Middelfart, Denmark; 3 University of Leeds, Woodhouse, Leeds, LS2 9JT UK; 4 Department of Child and Adolescent Psychiatry, Aarhus University Hospital, Palle Juul-Jensens Boulevard 175, 8200 Aarhus N, Denmark

**Keywords:** Restrictive practices, Psychiatric inpatient units, Children and adolescents, Mental health staff, Relatives, Systematic text condensation, Integrative review

## Abstract

Children and adolescents admitted to psychiatric inpatient units may be subjected to restrictive practices. Such practices can have significant psychological and physical consequences for the young inpatients, as well as for their relatives and the staff involved. Despite this, experiences of restrictive practices remain underreported particularly regarding the mechanisms that sustain their continued use, even in the face of international and national regulations aimed at reducing restrictiveness. A deeper understanding of these mechanisms can help nuance clinical decision-making and illuminate the complex relationships between staff, children, and relatives. This integrative review aims to provide such insights through a systematic literature search that includes both peer-reviewed and grey literature across quantitative and qualitative studies. The included papers were assessed using JBI and GRADE-CERQual and findings were analyzed using Systematic Text Condensation showing that restrictive practices persist not only due to acute crises but through intertwined mechanisms: their ethical justification in care, their institutional and legal normalization, and their embeddedness in everyday ward culture. Children and adolescents, relatives, and staff each shape how safety and responsibility are understood, reinforcing restrictive practices in different ways. These findings point to the need for strategies extending beyond regulation toward cultural and relational approaches that promote emotional safety, agency, and recovery—even when restrictive practices remain necessary. The review was preregistered at PROSPERO (CRD420251032437).

**Supplementary Information:**

The online version contains supplementary material available at https://doi.org/10.1186/s13034-026-01119-z.

## Introduction

Children and young people (CYP) are admitted into inpatient treatment to safekeep them during mental health crisis [1]. Restrictive practices are regularly used in some of these admissions to maintain safety, to protect the patient and/or others [2] and to manage challenging behavior otherwise unmanageable by less intrusive de-escalation techniques using i.e. restraint or seclusion [3]. Research is replete with varying definitions of practices that limit an individual’s autonomy, including terms such as restrictive interventions, coercion, and containment. In this paper, the term ‘restrictive practices’ (RP) is preferred and operationalized as “*any practice or intervention that has the effect of restricting the rights or freedom of movement of a person with disability, with the primary purpose of protecting the person or others from harm*” [4]. This definition is adopted as it captures the broad range of forms RP may take, including both deliberate and less deliberate actions enacted within asymmetric power relationships, where individuals with greater authority restrict the autonomy of those with less agency, often with the intention of preventing harm. Staff decision to use RP has been described in adult mental health settings as a dynamic and situated process shaped by ethical dilemmas, emotional responses, institutional constraints, and clinical judgment [5]. This understanding informs the present review’s synthesis of how restrictive practices are experienced and enacted across Child and Adolescent Mental Health Services (CAMHS).

Studies examining the prevalence of RP within CAMHS reveal substantial variations in reported rates and identify inconsistent, and sometimes conflicting, risk factors associated with their use [2, 6–9]. These findings suggest that RP cannot be fully explained by patient risk or safety considerations alone [6]. In response to such concerns, international and national bodies, including the World Health Organization and the Equality and Human Rights Commission, advocate for rights-based care and the minimization or elimination of RP [10, 11]. Various initiatives, i.e. Safewards [12–16], has been implemented in effort to remedy this with limited and short-lived success [2, 17] due to clinical and pathological insensivity [18].

One proposed pathway towards better clinical adaptation is to explore the perspectives of those directly involved in RP events [17]. Insight into how RP is experienced, justified, and contested by CYP, staff, and relatives may help explain why RP persists despite policy directives and reduction initiatives.

CYP report adverse psychological and physical consequences following the use of RP, including fear, humiliation, and loss of autonomy [19–21]. Simultaneously, some CYP experienced calming effect or increased safety following RP [22–25] highlighting its complex and ambivalent nature. Although research has documented how CYP perceives and reacts to RP, it rarely addresses their views on why RP happens, resulting in limited insight into the processes sustaining its use.

Staff perspectives further illustrate this complexity. While RP are often viewed by staff as necessary to ensure safety and regain control in crisis situations [26], their accounts reveal tension between institutional norms, legal frameworks, and professional therapeutic values [23, 27, 28] in some research summarized as moral distress [29]. Evidence from adult inpatient settings indicates severe psychological and physical consequences for staff following RP [30] contributing to poor staff retention [31] with substantial societal costs. Although research has explored how staff perceive and justify RP, it rarely examines their views on why RP is used, limiting insight into the factors that sustain its practice.

Relatives express similar mixed views, acknowledging perceived protective functions of RP alongside reports of trauma, exclusion from decision-making, and ethical concerns [23, 32, 33]. However, research specifically examining relatives’ experiences of RP in CAMHS is limited.

Although such perspectives are crucial for understanding the persistence of RP in inpatient CAMHS, the available evidence remains fragmented and insufficiently integrated across studies. This lack in integration reflects the contextual and relational complexity of RP, shaped by variations in institutional settings, professional roles, and care cultures. As a result, the mechanisms sustaining the use of RP remain under-theorized and dispersed across the literature rather than explicitly articulated. A comprehensive synthesis of these perspectives is therefore needed to clarify how RP are perceived, and which mechanisms may contribute to its continued use in inpatient CAMHS.

## Aim

This review aimed to explore and integrate the perspectives of CYP, relatives, and staff on the use of RP in CAMHS, and to examine the mechanisms contributing to its continued use.

## Method

This integrative review, conducted within a pragmatic realm [34], followed Whittemore and Knafl’s [35], consulting Toronto and Remington [36], five-stage framework and the PRISMA reporting guideline [37]. The protocol was prospectively registered in PROSPERO on 28 January 2025 (CRD420250644254). Following a PROSPERO website update, the administration requested the protocol to be re-registered with the same content; this was completed on 14 April 2025 and amended on 4th of March 2026 (CRD420251032437). Mainly the aim was refined during the review process to better reflect the available evidence and patterns identified in the included studies.

Papers were included if they examined experiences of RP among child and adolescent inpatients (< 18 years) currently under admission at a 24-h mental health ward, relatives, or staff in CAMHS and were published in English, Danish, Norwegian, or Swedish. No date or geographical limits were applied. The search was structured using the PICo framework (Population: staff/inpatients/relatives; Interest: broadly defined RP [4]; Context: CAMHS). Full search strategies are available in the PROSPERO supplementary file.

Searches were conducted on CINAHL (Ebsco), Embase Classic + Embase (Ovid), MEDLINE (Ovid), and APA PsycINFO (Ovid) on the 26th of February 2025 along with backward and forward citation searches [38]. Google Scholar and PAIS (ProQuest) were also used to uncover other relevant knowledge [36, 39, 40]. Grey literature was included if it met the same inclusion criteria as peer-reviewed studies, including a focus on experiences of restrictive practices in inpatient CAMHS settings and the provision of empirical or report-based data.

The search was updated on the 12th of January 2026. A university librarian assisted in retrieving papers if need arose and consulted the search strategy. Search terms are available in supplemental material. Data was extracted using the following data items: demographics (author(s), title, sample, RP definition and type, Scientific theory, and method) and experiences (from CYP, relatives, and staff).

### Quality and confidence assessment

All included studies, including peer-reviewed articles and grey literature, were appraised using Joanna Briggs Institute (JBI) critical appraisal tools appropriate to study design. During appraisal, it became evident that checklist-based ratings alone did not adequately convey overall methodological trustworthiness across the body of evidence. Therefore, a supplementary color-coded categorization was applied consistently across all included studies to summarize overall risk of bias. Studies categorized as green sufficiently met core quality criteria and demonstrated methodological coherence, with only minor issues related to reporting unclarity rather than missing or contradictory information, corresponding to very low risk of bias. Yellow indicated partial fulfillment of quality criteria, such as undeclared philosophical positioning or limited transparency, corresponding to moderate risk of bias. Red indicated substantial methodological shortcomings and high risk of bias. Importantly, limited reporting detail—particularly common in grey literature—was not in itself grounds for downgrading quality if other indicators of rigor and coherence were present.

Confidence in the review findings was assessed using GRADE-CERQual, informed by guidance from the JBI [41]. Each main analytical theme was assessed across four domains: methodological limitations, coherence, adequacy of data, and relevance [41–47]. The resulting confidence ratings (high, moderate and low) are reported in supplemental material and at the outset of each main theme to support transparent interpretation of the findings. It is important to note that the assessments do not indicate the importance of a theme, but the degree of confidence in the evidence underpinning it. Confidence assessments were applied to main themes rather than all sub-findings to avoid over-fragmentation and to maintain analytical coherence.

### Analytic approach

Systematic Text Condensation (STC) [48] was employed as analytical framework ensuring an a-theoretical approach. Authenticity markings were adapted to the STC approach to clarify analytical levels using ‘empirical headings’ (EH), ‘mixed headings’ (MH), and ‘analytical headings’ (AH) [49]. The analysis involved repeated readings of the studies included to ensure familiarity with the data. Relevant quotes and condensations were manually extracted by the first author and organized into a mind-map to facilitate an overview of patterns and emerging themes. This mind-map was continuously revised through an iterative process by the authors to ensure analytical depth throughout this analytical stage.

A reflexive approach was maintained throughout the analytical process [50, 51]. The first author, being a PhD student, has some experience with research in adult, forensic mental health and other fields relating to mental health, which may have shaped sensitivity to clinical processes and interpretations of mental health care contexts. The wider author team brought diverse professional perspectives, including experience from adult mental health and forensic mental health, as well as child and adolescent mental health. Notably, one co-author has a background as a nurse in child and adolescent mental health and several holds academic expertise’s within this field, contributing specific insight into the population under study.

These different disciplinary and clinical perspectives were actively engaged through ongoing discussions during the analytical and reviewing process to critically challenge interpretations and assumptions. This collaborative approach aimed to balance potential preunderstandings and enhance the credibility and trustworthiness of the manuscript by ensuring that findings were not shaped by a single professional lens.

## Results

**Fig. 1:**
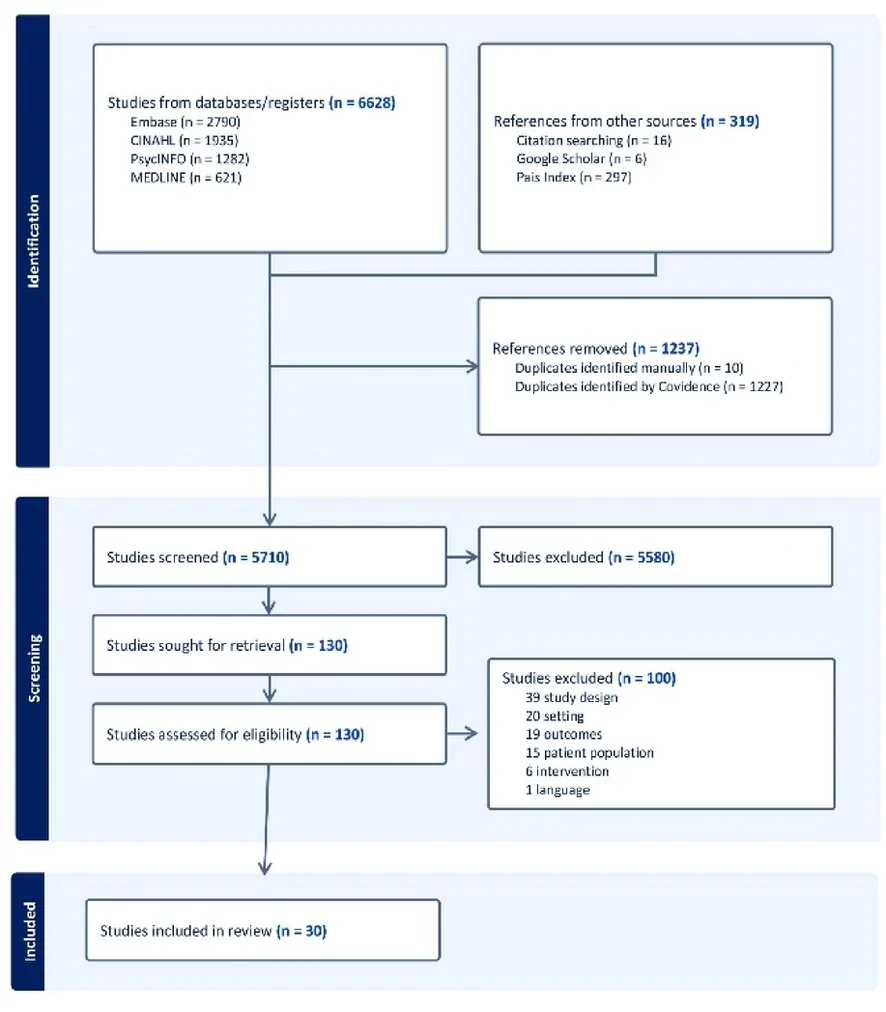
PRISMA flowchart of study selection

As depicted in Fig. 1: ‘PRISMA flowchart of study selection’ the search conveyed 6628 results from databases and other sources. Of these, 30 papers were included after removal of duplicates and screening processes. The results of the JBI assessment of each paper along with main findings are presented in Table S1 ‘Sample’. The included papers spanned across diverse geographical contexts, predominantly from the United States, United Kingdom, and Australia, with additional contributions from Europe, the Middle East, and South America. Terminology about RP varied across papers (e.g., restrictive interventions, restrictive practices, containment measures, coercion), and few provided explicit definitions, limiting conceptual clarity.

20 papers were included with a qualitative design. Nine of these held staff perspectives (n = 86) [26, 52–59]. Five papers held CYP perspectives (n = 17) [20, 60–63]. Three papers [64–66] included both staff perspectives (n = 30), relatives (n = 26) and CYP (n = 21) perspectives. Two studies of the same first author was an observational study of a 12 bed inpatient setting with staff and CYP interactions [67, 68] and one did not disclose [69].

Five papers were of quantitative design. Four papers focused primarily on CYP perspectives [19, 70–72] (n = 246) and one on staff (n = 128) [73].

Three papers consisted of a mixed-method approach. One held staff perspective (n = 55) [74] and one held CYP perspectives (n = 188) [75]. One study jointed staff (n = 81) and CYP (n = 81) perspectives [76].

Of the grey literature, one opinion article [77] held four staff arguments. One policy report was also found focusing on 56 CYP perspectives [78].

**Fig. 2:**
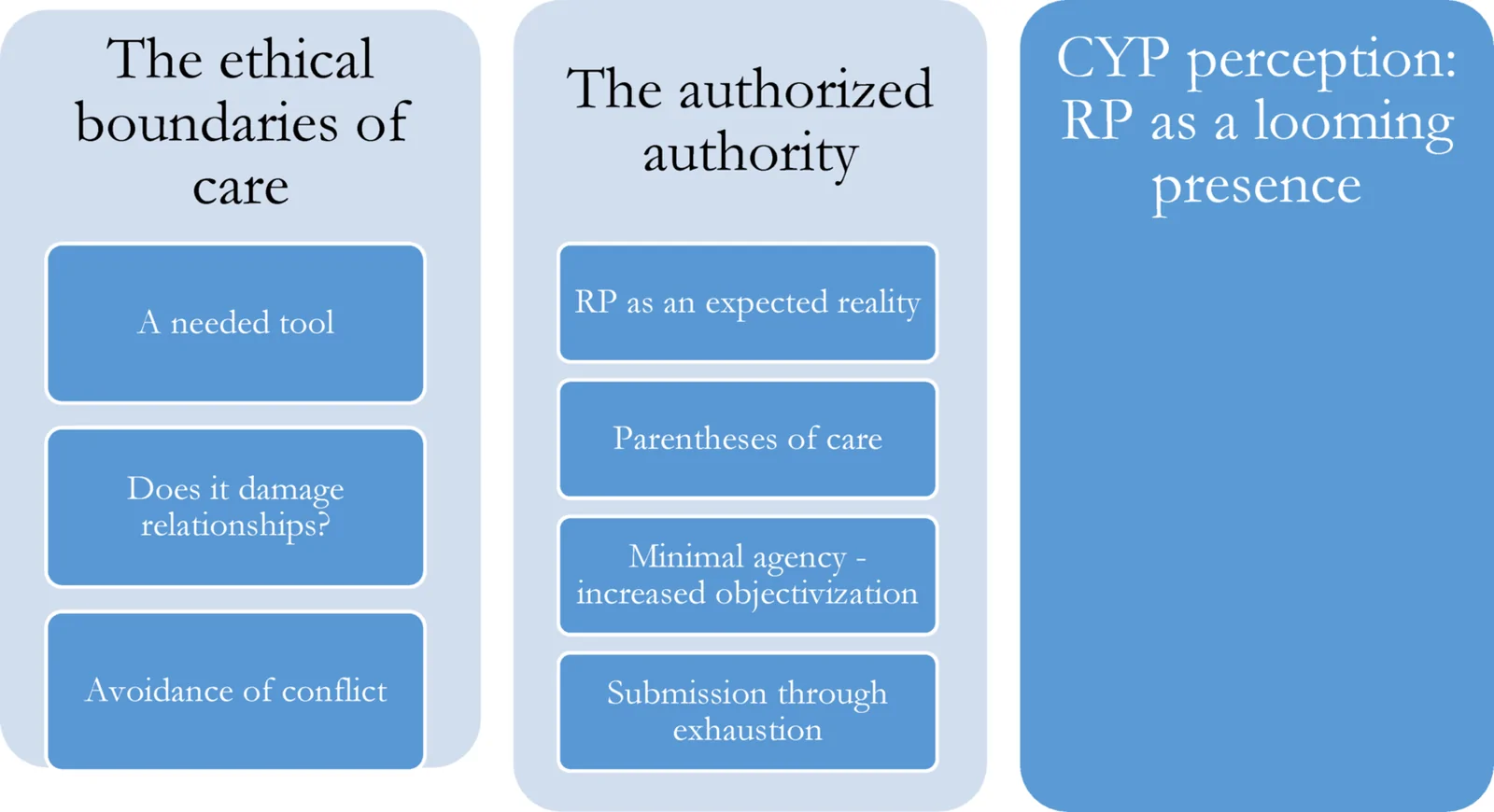
Results of the Thematic Analysis

The analysis revealed three main themes with supporting subthemes illustrated in Fig. 2: Results of the Thematic Analysis consisting of two overarching themes with subthemes and one theme without subthemes.

### The ethical boundaries of care: legitimizing RP in CAMHS

Staff often described RP as necessary to prevent imminent harm. One clinician noted: “*You watch that person banging their head on a corner of a wall and talk to them when they are completely out of control and they are completely unable to listen until they’ve split their skull wide open*.” [26]. Such accounts framed RP as an ethical duty rather than merely a clinical intervention, reflecting ongoing tension between protection, compassion, and regulation [79]. The theme along with the three subthemes were assessed with high confidence based on minimal methodological limitations, strong coherence across studies, and adequate data from relevant contexts.

#### A needed tool

Despite ethical debate, or maybe as a cause thereof, staff frequently viewed RP as essential in acute crises and sometimes beneficial for maintaining safety [26, 57, 59, 76, 77]. As one staff member noted: “*People who talk about moving away from restraint completely have never worked on a children and adolescent mental health ward”* [26]. RP was described as “*not great, but*…” [59] signifying a (reluctant) acceptance of its necessity [53]. Staff acknowledged using RP pragmatically as a quicker alternative to de-escalation, a way to influence behavior, or sometimes as “*the easy way out*” [55]. Thus, protective reasoning often merged with practical considerations such as efficiency and workload.

#### Does it damage relationships?

Some staff believed strong therapeutic relationships buffered RP’s negative impact. One explained: “*No matter what they do, it’s not going to change how I am with them, so even though I might have had to restrain them minutes ago, the relationship hasn’t changed, and I make sure they know that* […]*.*”[64]. This perspective, echoed in other studies [26, 71], suggested that RP was not universally perceived as harmful within CAMHS. Some staff framed RP as respectful, empathic, and caring when embedded in a trusting relationship [58, 64]. Similarly, some CYP legitimized RP [62, 64, 70, 73], particularly when relatives endorsed it [66, 70, 80] (connecting to the theme “Parentheses of care: Legal relief and emotional responsibility”). Yet other staff described RP as emotionally draining and potentially harmful: “*It’s scary, it’s emotionally draining for both the patient and staff... there’s blood coming out [from the young person’s nose], the child is screaming down the place, so as much as you’re supporting the child, it becomes very difficult because it seems like you’re either attacking or physically punishing somebody*” [53]. This perspective illustrates staff’s sense of causing harm which for some resembled punishment [64] rather than providing care.

#### Avoidance of conflict

Some staff described the emotional toll as overwhelming, prompting strategies to detach from their feelings: “*I am sure I am having emotions. But I wouldn't say most the time I'm aware of them. I try to block the emotions*.” [56] suggesting staff vicarious trauma [26, 57, 59]. To mitigate emotional strain, some staff avoided escalation by refraining from “*pushing the buttons*” [58] or walked away when CYP defied instructions [67, 68]. These responses signaled resignation or conflict avoidance; a strategy that reduced immediate trauma exposure but carried relational risks, as CYP sometimes interpreted withdrawal as abandonment [20] creating further tensions possibly leading to delay conflict until situations become critical.

### The authorized authority

Legislative reforms did not consistently reduce RP; two studies found unchanged practice following policy changes [54, 69]. This suggests that law both restricts and legitimizes RP, embedding it within institutional norms [55] (relates to the theme: A needed tool). The theme and its four subthemes were assessed with moderate methodological and coherence concerns but minor or no concerns regarding adequacy and relevance.

#### RP as an expected reality

RP sometimes shifted from crisis-specific to habitual as past RP episodes also shaped some future choices [69]. A staff reflected: “*One time might be okay, but not a second time, because then it’s like, it’s hard to turn back once you’ve started. Twice becomes a habit, you know*” [55]. Furthermore, the analysis showed that legal frameworks tended to reinforce staff expectation; Staff appeared to internalize the legal framing, treating RP as an accepted and expected part of care [55, 57]. Systemic pressures—including understaffing and high-demand periods—were linked to increased RP use [69, 74]. In these situations, RP was often initiated to maintain safety when systemic pressures left staff without the time or resources to implement less intrusive strategies. Staff’s arguments to use RP often reflected their expectations and the realities of their work environment, but staff and CYP alike reported confusion about the application and rules governing RP (relates to the theme: Ward atmosphere) [54, 72, 74, 81] suggesting inconsistencies between policy and practice.

#### PARENTheses of care: legal relief and emotional responsibility

The legal framework enabled relatives to admit CYP to CAMHS when care became unmanageable, effectively transferring legal, ethical and practical feelings of responsibility to staff [60, 64, 66, 78]. This shift often provided relief but also heightened expectations: “[…] *he's meant to be with you, and you're meant to be trying to keep him safe and you're not*.”[65]. Relatives frequently sought earlier intervention than staff deemed appropriate, creating tension [65, 74]. This mismatch may reflect differing perceptions of risk and responsibility, where families, often closer to CYP’s distress, may prioritize immediate action to prevent harm, while staff rely on clinical thresholds and procedural frameworks as alternative strategies to RP. Some papers noted parents’ mixed relief and guilt when RP was used, especially during involuntary feeding [66]. Staff sometimes used relatives’ involvement to encourage CYP compliance [62] and relatives’ endorsement reduced perceived coercion among CYP [70].

#### Minimal agency—increased objectivization: CYP internalize institutional control

RP often left CYP feeling objectified and dehumanized [20, 60, 61, 64, 78] (relates to the theme: Does it damage relationships?), but was also internalized by CYP feeling “*disempowered, confused, and controlled by others*” [79]. Loss of bodily autonomy was experienced as degrading [61] and invoked “*feelings of a lack of identity*” [81]. In one study examining adolescents’ perceptions of seclusion and restraint, 40.4% of patients who had never been secluded were unaware that seclusion could be used, and 88.7% reported that they had not been informed about this possibility. This lack of knowledge appeared to contribute to fear, particularly when CYP heard the reactions of other inpatients during seclusion [79]. Conversely, when staff explained the rationale for RP, CYP reported greater understanding and engagement [62]. RP appeared particularly coercive among CYP with eating disorders amplifying vulnerabilities such as shame and loss of control [70]. Such experiences may heighten feelings of powerlessness and contribute to patterns of submission, where apparent calmness reflects diminished agency rather than genuine emotional regulation.

#### Submission through exhaustion

Some CYP preferred seclusion to restraint [19, 59] particularly when staff remained present [79]. Some studies suggested that seclusion could calm CYP [19, 78], but one noted this may mirror emotional exhaustion rather than true regulation [74]. This indicates that RP may reduce overt resistance but not without emotional cost. Importantly, what staff interpreted as calmness may have reflected emotional exhaustion or fear-based submission rather than genuine emotional regulation (relates to the theme: Does not damage relationships?). Staff accounts such as: “[…] *let's put him in the multi-purpose room* […]*, he will be fine then, he will just fall asleep from exhaustion*” [20] reinforce this interpretation, which is elsewhere described as giving up a fight [81] and as a resistance phase [71]. Thus, cessation of resistance may signal exhaustion rather than therapeutic success. Furthermore, resistance—sometimes linked to previous trauma reenacted by RP [52]—is subtly presented in some papers as a rationale for RP use [57, 60, 67, 68, 78].

### CYP perception: RP as a looming presence

A smaller but analytically important finding suggest that RP was experienced and valued as more than the actual RP form; RP appeared to be intrinsically intertwined with the ward atmosphere [62] (relates to the theme: RP as an expected reality). Although supported by fewer studies and assessed with moderate concerns regarding methodology, adequacy, and relevance, this finding highlights a distinctive experiential dimension of RP that extends beyond discrete interventions.

RP was experienced as more than a singular event; its possibility seemed to permeate the ward atmosphere (relates to the theme: The Ethical Boundaries of Care: Legitimizing RP in CAMHS). Nielson [62, 81] described this anticipatory element as *“Forecasting the Thunderstorm”*, where CYP sensed RP as a looming presence even before it was enacted: “[…] *they think I don't know what's about to happen … it's not a secret … it's obvious when it will happen*” [81]. This anticipation shaped emotional safety and behavior, suggesting that authority was exercised not only through actual RP but also through its symbolic availability [78] seemingly permeating everyday interactions [57, 60, 67, 68, 78]. For CYP, this anticipatory dimension appeared to amplify relational mistrust and behavioral self-monitoring, rendering even routine interactions subject to interpretation through a lens of potential restriction [62, 65, 79] In contrast to adult inpatient accounts, where RP is more often described as episodic or event-bound, CYP experiences suggested a more diffuse and atmosphere-driven form of control, potentially reflecting adolescents’ heightened sensitivity to adult authority, dependency, and ongoing identity formation [20, 70].

### Synthesis of findings

To further illustrate the integration of perspectives across CYP, relatives, and staff, Table 1 synthesizes how restrictive practices are conceptualized and experienced across the three analytical themes. Rather than presenting these perspectives as isolated, the table highlights their relational positioning within shared care contexts, illustrating how interactions between CYP, relatives, and staff may contribute to the normalization and persistence of restrictive practices.

**Table 1:** Synthesization of findings

Theme	Staff perspectives	CYP perspectives	Relatives’ perspectives
The Ethical Boundaries of Care: Legitimizing RP in CAMHS	RP framed as necessary, protective, and ethically justified in acute crises; described as a “needed tool” despite discomfort; sometimes viewed as caring and relationally buffered when trust is present; also acknowledged as emotionally draining and occasionally resembling punishment; at times used pragmatically (efficiency, workload)	Experiences range from abhorrent and dehumanizing to occasionally understood or legitimized, especially when embedded in trusted relationships or supported by relatives; some experience calmness, others fear, confusion, and loss of control	Mostly absent, but when present, relatives may indirectly contribute to legitimizing RP through support or acceptance, influencing CYP perceptions
The Authorized Authority	RP embedded within legal and institutional frameworks, reinforcing its position as expected and normalized; shaped by system pressures (staffing, workload); may shift from crisis response to habitual practice; staff report confusion about rules. Staff view RP through the shared responsibility, with relatives’ involvement sometimes used for CYP compliance	Often experience RP as dehumanizing, objectifying, and exhausting; may show submission or compliance due to exhaustion rather than true calm; sometimes prefer less intrusive forms (e.g., seclusion with staff presence)	Experience relief from transferred responsibility, but also guilt and emotional burden, especially when RP (e.g., feeding) is used; may both support and question interventions. Otherwise limited visibility, but indirectly involved through legal authority transfer when admitting CYP; contribute to the broader system legitimization of care structures
RP as a looming presence	RP normalized as part of everyday care and embedded in ward routines	RP experienced as a pervasive and anticipatory presence (“forecasting the thunderstorm”), shaping behavior, emotional safety, and trust	Rarely explicitly described but is experienced as a sign of care, possibly embedded within expectations of staff profiles, possibly contributing to the formation of an RP-atmosphere

As shown in Table 1, staff perspectives predominantly emphasized safety, necessity, and institutional constraints, whereas CYP and relatives more often highlighted experiences of distress, reduced agency, and emotional ambivalence, thereby challenging and contextualizing staff rationalizations.

Viewed collectively, the synthesized findings suggest that restrictive practices are sustained through interconnected dynamics between CYP, relatives, and staff. Relatives’ endorsement or acceptance of RP may legitimize staff decision-making and ease ethical tension, while staff reliance on institutional authority can shape CYP experiences of control, compliance, and internalized regulation. These relational processes may form self-reinforcing cycles within ward culture, contributing to the normalization of RP as part of ward atmosphere.

## Discussion

Our findings identified several mechanisms that appear to contribute to the sustained use of RP in CAMHS. Across CYP, relatives, and staff accounts, three mechanisms consistently emerged: (1) ethical legitimation within care, (2) institutional and legal reinforcement, and (3) RP’s symbolic presence within the ward climate. Together, these mechanisms provide an explanatory framework for the persistence of RP despite widely documented policy initiatives, clinical guidelines, and organizational strategies aimed at reducing their use, as described in the Introduction. While such reduction efforts primarily target formal procedures and decision thresholds, the present synthesis highlights how ethical reasoning, institutional normalization, and relational dynamics may continue to reproduce RP in everyday practice. The three mechanisms are discussed in the following two paragraphs, with the third highlighting parental involvement as a particular area of ethical and relational complexity.

### Ethical legitimation and ward culture

Staff frequently framed RP as necessary, pragmatic, and sometimes caring, especially when used within perceived trusting relationships, which has been highlighted in similar research on staff working in adult mental health settings [82]. This ethical framing—accurate or not—lessens the perceived imperative to reduce RP and positions it as a morally acceptable and clinically responsible option. Developmental views of CYP may contribute to this. Montreuil [83] argues that CYP are often positioned as “*incomplete human becomings*” reinforcing paternalistic assumptions. Such framing can embed RP within ward culture as a subtly validated response, consistent with CYP reports of RP as a constant symbolic presence.

Within this developmental lens, RP can appear legitimate for managing dysregulation, yet this same framing risks obscuring relational or traumatic origins of CYP distress. Trauma can disrupt neurodevelopment [84] and extensive evidence documents the psychological harms associated with RP [19–21]. Importantly, recovery in young people is understood as a relational, non-linear process shaped by identity development, agency, and social context, rather than simply symptom reduction [85, 86]. In this light, RP undermine agency or relational trust which may interfere with core recovery processes [87]. These insights underscore the need for staff to reflect on their use of RP and the developmental assumptions shaping their decisions.

### Institutional and legal authority: disruption of identity

One important finding in the review was the complexities of RP experiences within CAMHS; some CYP found the practices abhorrent, but some did find comfort and calmness following RP. This ambivalence is echoed in previous research, where some inpatients perceived certain aspects of RP as more beneficial than harmful [88, 89]. These findings may help explain the continued use of RP in clinical practice. In acute situations, such as when individuals are at risk of acting on suicidal thoughts or displaying severe aggression towards others, RP may serve as immediately protective and stabilizing. Thus, evidence supporting the short-term efficacy of RP in managing acute risk cannot be completely disregarded. However, this does not negate the substantial body of evidence demonstrating their longer-term psychological and relational harms. Importantly, these findings should not be skewed in either direction; RP cannot be understood as solely harmful or beneficial. Rather, they represent a paradoxical intervention that may, in specific circumstances, contribute to immediate safety, while simultaneously carrying the risk of longer-term adverse consequences. This is important since the magnitude and consistency of these beneficial and harmful effects remain unclear due to the limited quantitative evidence available.

Previous qualitative studies have shown that RP can disrupt recovery through negative psychological and relational impacts [30, 90–92]. CYP frequently reported feelings of dehumanization in this review, a concern also well documented in adult mental health settings [88], reflecting experiences of objectification and loss of agency. Beyond potential neurodevelopmental consequences, diminished self-worth may further increase vulnerability to self-destructive behaviors [93, 94]. When CYP internalize institutional authority, emotional resources may shift from recovery and towards resistance, which may result in aggressive behavior towards self/others; behaviors that often precede the use of RP [3, 16, 21, 81]. This dynamic may contribute to a self-reinforcing cycle in which RP, while intended to restore safety, also sustains or exacerbates the distress it seeks to manage. Over time, such cycles may undermine identity development, trust, and engagement with care, further complicating clinical and recovery-oriented trajectories.

As such, RP should not be viewed as a one-sided phenomenon. Research on staff decision-making describes the use of restraint as “*a dynamic process shaped by ethical dilemmas, negotiations between professional ideals and clinical necessity, institutional constraints, and lived emotions*” [5]. This review extends this conceptualization by demonstrating how parents’ involvement within CAMHS, and their decision-making processes and influence, may be equally important within CAMHS. From the perspective of CYP, staff decision-making may also encompass informal practices, such as access to parents, thereby blurring the boundaries between formal restriction and relational control. Consequently, decisions to use RP appear influenced by organizational structures, relational dynamics, and emotional staff responses, extending beyond immediate safety concerns alone.

### Relatives’ and relational-legal legitimation

Relatives’ perspectives both mitigated and reinforced RP. Many advocated early interventions for self-harm yet felt guilt or blame when RP was used. Such conflicting expectations created moral tension for staff, who felt responsible both to families and institutional safety mandates. The framing of RP as a form of care aligns with findings in psychosis care [33]. Effective collaboration depends on transparent communication [95] and relatives’ alignment with staff decisions supports treatment engagement [1, 96]. Furthermore, joint participation of parents, staff and adolescents has been found to elevate and strengthen the therapeutic process [97, 98]. Similar research within adult mental health echoes this, showing how carers/parents wish to be included from admission to discharge to ensure effective reduction or elimination of seclusions and restraints [99]. However, staff sometimes used relatives’ involvement to encourage CYP compliance, thereby legitimizing RP through relational as well as institutional channels. This can border on informal coercion [55, 100, 101] raising ethical concerns and may explain why CYP experience staff authority as permeating the ward atmosphere, whereby control is exercised not only through enacted RP but also through interactions not explicitly intended as such.

For CYP—whose attachment systems are heightened in crisis—the perception that staff and relatives jointly sanction RP may amplify feelings of betrayal or abandonment. Such experiences shape internal working models and may contribute to insecure attachment patterns [102]. This is particularly relevant in settings where care is simultaneously protective and coercive and where CYP’s developmental futures are at stake.

### Implications for clinical practice and future research

The findings of this review highlight that RP within CAMHS are sustained not only through formal decision-making and institutional frameworks but also through relational, ethical, and symbolic dynamics embedded in everyday ward life. Clinically, this underscores the need for interventions that extend beyond procedural reduction strategies to address ward culture, relational trust, and the informal exercise of authority, including staff–parent dynamics and to insert policy, training and adequate staffing supporting this.

Future research should examine how these relational and institutional dynamics interact with more complex clinical presentations over time, particularly in cases involving repeated use of RP. Longitudinal and ethnographic approaches may be especially valuable for capturing how RP become normalized within ward climates and how CYP developmental, relational, and emotional vulnerabilities shape their experiences differently from adult inpatient populations. Research that explicitly adopts a triadic perspective—including CYP, relatives, and staff—may further illuminate how RP are negotiated, legitimized, and reproduced in clinical practice.

### Limitations

This review drew primarily on qualitative studies, supplemented by quantitative and mixed-methods work, which may limit generalizability. Although an integrated design suited the study aim, combining qualitative and quantitative evidence requires methodological caution [103]. Most included studies were conducted by nurses or psychologists as first authors, introducing potential sampling or interpretive bias [104]. GRADE-CERQual assessments were conducted by a novice researcher, which may introduce novice-level bias despite efforts to ensure rigor through authorship cooperation.

Although a standardized appraisal approach was applied across all studies, assessing overall trustworthiness required interpretive judgment, particularly in the context of heterogeneous reporting standards.

The synthesis relied on literature centered on CYP, relatives, and staff perspectives, with fewer studies explicitly addressing mechanisms sustaining RP. Policymaker perspectives were limited, and most studies originated from Western contexts, restricting cultural transferability. Including older publications may have introduced temporal variability, though more recent studies largely corroborated these findings. Variation in RP terminology also complicated synthesis and required careful interpretation. While thematic analysis is well suited to explore experiences, its interpretive nature inevitably contains subjectivity. Finally, the review reflects the perspectives of those directly involved in RP but does not capture broader organizational or systemic determinants.

## Conclusion

This integrative review finds that the continued use of RP in CAMHS cannot be understood solely as a response to acute crises. Instead, CYP, relatives’ and staffs’ accounts indicate that RP may be sustained through a constellation of interconnected mechanisms—including its ethical legitimation within care, its normalization through institutional and legal authority, and its symbolic presence within everyday ward culture and relationships. Staff, relatives, and CYP each contribute to these dynamics in different ways, shaping how safety, responsibility, and intervention are understood in practice. Recognizing these mechanisms highlights the need for research and strategies that extend beyond regulatory change toward cultural, relational, and developmental understanding and approaches that support emotional safety, agency, and recovery for all those involved—even in situations where RP may remain warranted.

## Supplementary Information

Below is the link to the electronic supplementary material.

Supplementary Material 1: Sample

Supplementary Material 2: GRADE_CERQual

## Data Availability

Search strings, along with search terms etc., are available online through the PROSPERO (CRD420251032437). Models illustrating the analytical process are available per reasonable request to the corresponding author. Tables and PRISMA figure are available in supplemental materials.

## Abbreviations

RP: Restrictive practices
CAMHS: Child and Adolescent Mental Health Settings
CYP: Children and Young People
STC: Systematic Text Condensation
GRADE-CERQual: Grading of Recommendations Assessment, Development and Evaluation-Confidence in the Evidence from Reviews of Qualitative Research
CINAHL: Cumulative Index to Nursing and Allied Health Literature 1976–2026 (Ebsco)
EMBASE: Excerpta Medica dataBASE 1947–2026 (Classic + Embase (Ovid))
MEDLINE: MEDical Literature Analysis and Retrieval System Online 1946–2026 (Ovid)
PAIS: Public Affairs Information Services 19,152,026 (ProQuest)
APA PsycINFO: American Psychological Association PsycINFO 1806–2026 (Ovid)
PROSPERO: Prospective Register of Systematic Reviews
JBI: Joanna Briggs Institute
PRISMA: Preferred Reporting Items for Systematic Reviews and Meta-Analyses
